# Pyroptosis patterns of colon cancer could aid to estimate prognosis, microenvironment and immunotherapy: evidence from multi-omics analysis

**DOI:** 10.18632/aging.204302

**Published:** 2022-09-23

**Authors:** Jing Zhou, Hao Guo, Likun Liu, Mali Feng, Xihua Yang, Shulan Hao

**Affiliations:** 1 Department of Oncology, Shanxi Province Academy of Traditional Chinese Medicine, Shanxi Province Hospital of Traditional Chinese Medicine, Taiyuan, Shanxi 030012, China; 2 Shanxi Clinical Research Center of Traditional Chinese Medicine Affiliated Shanxi Hospital of TCM, Taiyuan, Shanxi 030012, China; 3 Department of Anesthesiology, Shanxi Provincial People’s Hospital, Taiyuan, Shanxi 030000, China; 4 Central Laboratory, Shanxi Province Academy of Traditional Chinese Medicine, Taiyuan, Shanxi 030012, China; 5 Affiliated Cancer Hospital, Shanxi Medical University, Taiyuan, Shanxi 030013, China

**Keywords:** colon cancer, tumor microenvironment, pyroptosis patterns, immunotherapy, The Cancer Genome Atlas

## Abstract

Pyroptosis plays a critical role in the occurrence and development of colon cancer (CC). However, the specific mechanisms of pyroptosis patterns on immune regulation and tumor microenvironment (TME) formation in CC remain unclear. Based on 30 pyroptosis-related genes (PRGs), we evaluated the pyroptosis patterns of 1689 CC samples from the Cancer Genome Atlas and the Gene Expression Omnibus databases. The signatures of pyroptosis patterns and PRGs were identified in CC. In addition to systematically associating these patterns with TME cell infiltration characteristics, we constructed a pyroptosis signature score (PPSscore) to quantify pyroptosis patterns in individual tumor patients with immune responses. We discovered three distinct pyroptosis patterns, each with a different survival probability and being biologically relevant. TME infiltrating characteristics of revealed these patterns, consistent with immune-inflamed, immune-desert and immune-excluded phenotypes. Furthermore, a low PPSscore was associated with better clinical benefits. A high PPSscore was associated with a lower chance of survival due to its association with stromal activation. Additionally, two immunotherapy cohorts revealed that patients with lower PPSscore had better immune responses and durable clinical benefits. Our findings indicate that pyroptosis patterns play a vital role in immunoregulation and the formation of TME in CC.

## INTRODUCTION

Pyroptosis, a new form of programmed cell death [1, 2], differs from apoptosis in terms of its ability to elicit a strong inflammatory response and its characteristic morphology of forming plasma membrane pores [3]. It has garnered increasing attention in recent years. When stimulated, the pattern recognition receptors in the cell act as receptors to recognize these signals and trigger pyroptosis. The assembly and activation of inflammasomes are crucial. It then activates the inflammasome-activated caspase-1 and intracellular lipopolysaccharide activated caspase-11/4/5. The activated caspases then cleave gasdermin D (GSDMD) at the _272_FLTD_275_ site, removing the inhibitory GSDMD-C domain and contributing to N-terminal oligomerization in membranes to form pores [4–6], resulting in lytic cell death and the release of inflammatory cytokines [7, 8]. The inflammatory cascade is triggered by the release of pro-inflammatory mediators into the extracellular environment. Pyroptosis was first discovered in the immune defense against bacterial infection [9]. Accumulating evidence reveals that it also plays a crucial role in developing other diseases, particularly cancer, the leading health menace in recent decades.

Despite advances in therapeutic strategies, the five-year overall survival (OS) rate for colon cancer (CC), one of the most common cancers worldwide, remains relatively low [10]. Scientists have shifted from simply focusing on tumor cell inheritance to realizing that the integral tumor microenvironment (TME) is widely implicated in tumorigenesis. TME, in addition to tumor cells, contains noncancer cell types (such as stromal cells, infiltrating immune cells, endothelial cells, and others.) and extracellular components (blood vessels, secreted cytokines, extracellular matrix, and others.) [11]. All these components contribute to the malignant phenotypes of cancer and immune escape. Aside from the genomic aberrations, tumor cells’ therapeutic responses also rely on the composition of the TME, which may significantly influence the clinical outcomes. Immunotherapy is the focus of tumor research at present. CC patients who respond to immunotherapy, particularly immune checkpoint inhibitors (ICIs), show a durable response [12, 13]. Though effective as it can be, the patients who exhibit dramatic responses is only occupy a small proportion. Previous research identified three basic immune profiles: the immune-inflamed, the immune-desert and the immune-excluded phenotypes, representing different TME characteristics and therapeutic options [14–16]. Thus, using TME to assess immune infiltration is vital for predicting existing ICI responses and developing new immunotherapy strategies [16–18].

The occurrence of pyroptosis has been shown to influence the TME antitumor immune response [19]. CD8^+^ T cells and NK cells released granzymes that could cleave GSDMB/E and thus triggered tumor cell pyroptosis, indicating that pyroptosis might play a role in anti-tumor immunity [20, 21]. Moreover, a GSDMD deficiency reduced the cytolytic capacity of CD8^+^ T cells in the vicinity of immune synapses [22]. Evidence also shows that the inflammatory factors released by pyroptosis may initiate an inflammatory cascade that influence tumor immunity. Hence, comprehensive characterizations of TME cell infiltration mediated by multiple pyroptosis patterns will improve our understanding of TME immune mechanisms.

In this study, we combined transcriptome and genomic data from 1689 CC samples from the Cancer Genome Atlas (TCGA) and the Gene Expression Omnibus (GEO) databases to comprehensively evaluate the association between pyroptosis patterns and TME cell infiltration characteristics and identified three distinct pyroptosis patterns. We also investigated the TME characteristics of these distinct patterns [14]. Furthermore, we constructed a pyroptosis signature score (PPSscore) to predict clinical response to ICI treatment and quantify pyroptosis patterns for individual patients. These findings indicated that pyroptosis patterns played vital roles in forming the diversified TME of CC and that they might be indispensable in guiding therapeutic interventions for CC.

## RESULTS

### The landscape of variation of pyroptosis-related genes (PRGs) in CC

**Figure 1:**
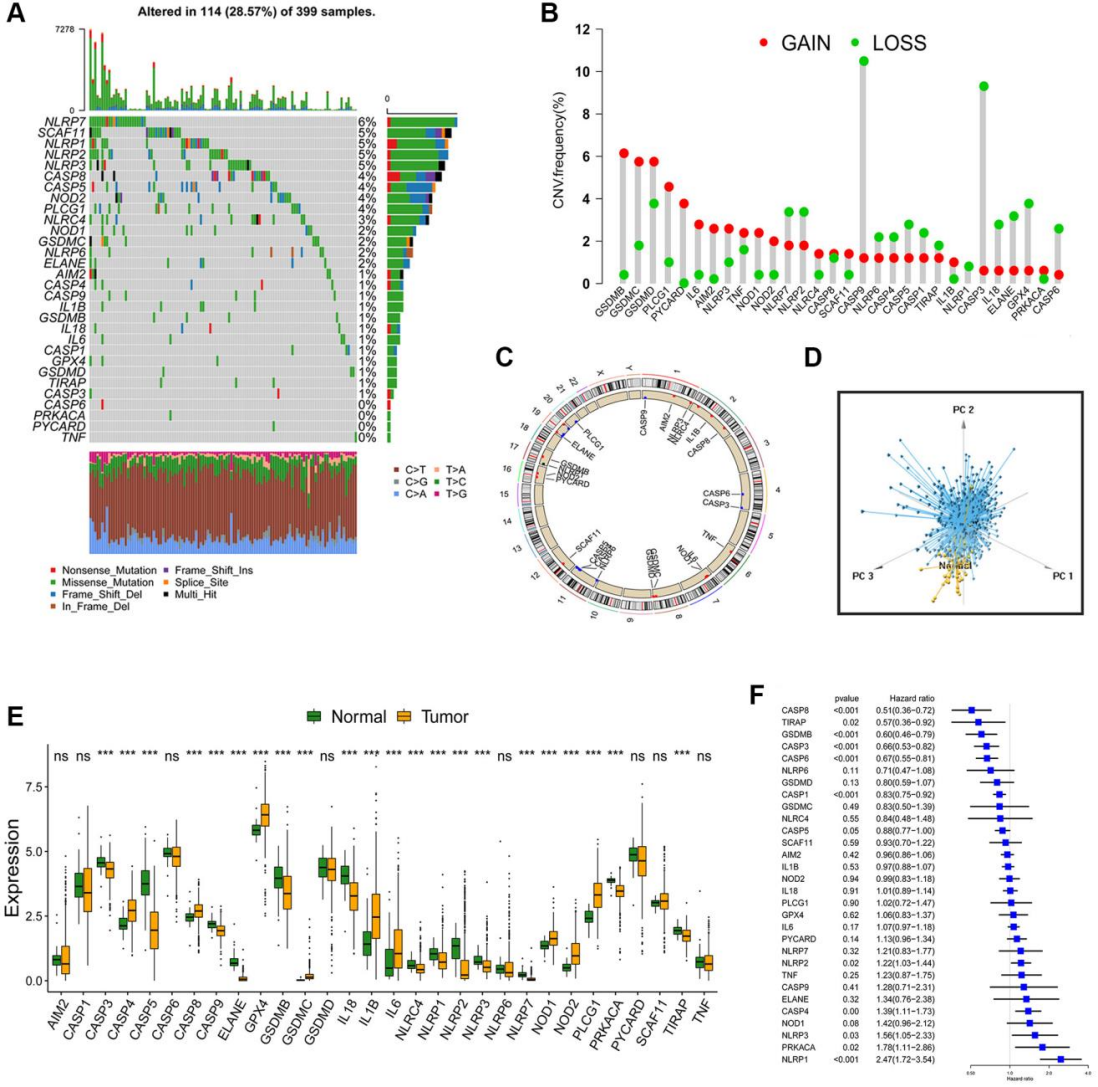
**Landscape of genetic and expression variation of PRGs in CC.** (**A**) Total of 114 of the 399 CC patients experienced genetic alterations of PRGs, with a frequency of 28.57%. The upper barplot showed the tumor mutational burden. The number on the right indicated the mutation frequency in each gene. The stacked barplot below showed fraction of conversions in each sample. Each column represented every individual patient. (**B**) The histogram showed the CNV variation frequency of PRGs. The height of the column represented the alteration frequency. The deletion frequency, green dot; The amplification frequency, red dot. (**C**) The location of CNV alteration of PRGs on 23 chromosomes. (**D**) Principal component analysis for the expression profiles of 30 PRGs to distinguish tumors from normal samples. Tumors were marked with blue and normal samples were marked with yellow. (**E**) The difference of mRNA expression levels of 30 PRGs between normal and CC samples (^*^P < 0.05; ^**^P < 0.01; ^***^P < 0.001). (**F**) The univariate Cox regression model was used to analyze the prognosis of 30 PRGs in 6 CC cohorts. Hazard ratio >1 indicated risk factors for survival, and hazard ratio <1 indicated protective factors for survival.

Finally, 30 PRGs, (AIM2, CASP1, CASP3, CASP4, CASP5, CASP6, CASP8, CASP9, ELANE, GPX4, GSDMB, GSDMC, GSDMD, IL18, IL1B, IL6, NLRC4, NLRP1, NLRP2, NLRP3, NLRP6, NLRP7, NOD1, NOD2, PLCG1, PRKACA, PYCARD, SCAF11, TIRAP and TNF) were identified. First, we examined the profile of PRGs copy number variations (CNV) and somatic mutations in the CC sample. The results revealed that CNV alteration is common in the 30 PRGs. As shown in Figure 1A, 114 samples experienced mutations of PRGs (the total number is 399, with a frequency of 28.57%). NLRP7 and SCAF11 genes had the highest mutation frequency, while no mutation was detected in the CC sample’s CASP6, PRKACA, PYCARD and TNF genes. The number of genes with copy number amplification was roughly equal to the number of genes with a widespread frequency of CNV deletion (Figure 1B). The location of CNV alteration of these PRGs on chromosomes was shown (Figure 1C). We could easily select the cancer samples based on the above characteristics (Figure 1D). Following that, we examined the mRNA expression levels of these genes. Tests were performed between normal and CC samples to determine whether the expression of PRGs in CC patients could be affected by the above-mentioned genetic variation. PRGs with amplified CNV had significantly higher expression in CC tissues (such as GSDMC and PLCG1) than in normal colon tissues (Figure 1B and 1E). These findings strongly suggest that changes in CNV may be substantial cause for the change of PRGs expression. Comprehensive analysis of the above, distinguishing normal and CC samples from CNV changes to final genetic and expression changes, revealed that the imbalance of PRGs expression played a key role in the progression of CC.

### Pyroptosis patterns mediated by 30 PRGs

**Figure 2:**
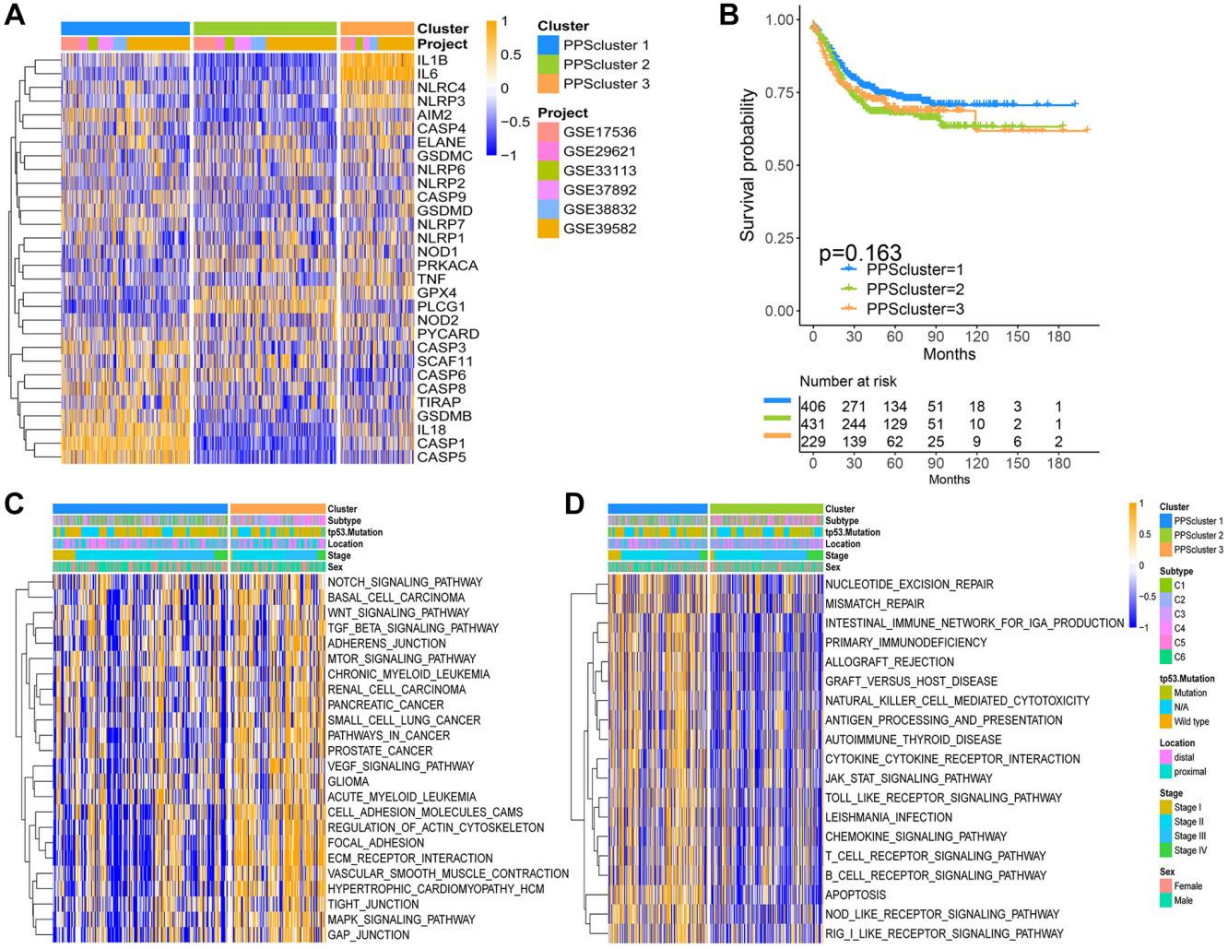
**Pyroptosis patterns and relevant biological pathway for each pattern.** (**A**) Unsupervised clustering of 30 PRGs in the six CC cohorts. The PPSclusters and cohorts’ names were used as patient annotations. Each column represented patients and each row represented PRGs. (**B**) Unsupervised clustering analysis of CC patients from 6 GEO cohorts (GSE39582, GSE38832, GSE37892, GSE33113, GSE29621 and GSE17536) resulted in three pyroptosis patterns. Kaplan-Meier curves of relapse-free survival for CC patients in the meta-GEO cohort with different pyroptosis patterns. (**C**, **D**) The heatmaps were used to visualize the gene set variation analysis score of representative biological pathways in distinct pyroptosis patterns. The color of orange represented activated pathways and blue represented inhibited pathways. The CC cohorts were used as sample annotations. PPScluster-1 vs. PPScluster-3 (**C**) and PPScluster-1 vs. PPScluster-2 (**D**).

Six GEO datasets (GSE39582, GSE38832, GSE37892, GSE33113, GSE29621 and GSE17536, Supplementary Table 1) were enrolled into one meta-cohort. A univariate Cox regression analysis was used to determine the relationship between the 30 PRGs and the prognosis of CC patients. The forest plot revealed that CASP8, TIRAP, GSDMB, CASP3, CASP6, CASP1 and CASP5 genes could be considered protective factors, whereas NLRP2, CASP4, NLRP3, PRKACA and NLRP1 genes could be considered risk factors (Figure 1F). The findings revealed the prognostic values of 30 PRGs in CC patients. Then, we classified different pyroptosis patterns based on the expression of 30 PRGs. Using unsupervised clustering, we identified pattern 1 (PPScluster-1), pattern 2 (PPScluster-2) and pattern 3 (PPScluster-3) (Figure 2A). Prognostic analysis of the three distinct patterns revealed that the PPScluster-1 pattern had a survival advantage (Figure 2B).

### The pyroptosis patterns characterized by distinct immune cell infiltration

**Figure 3:**
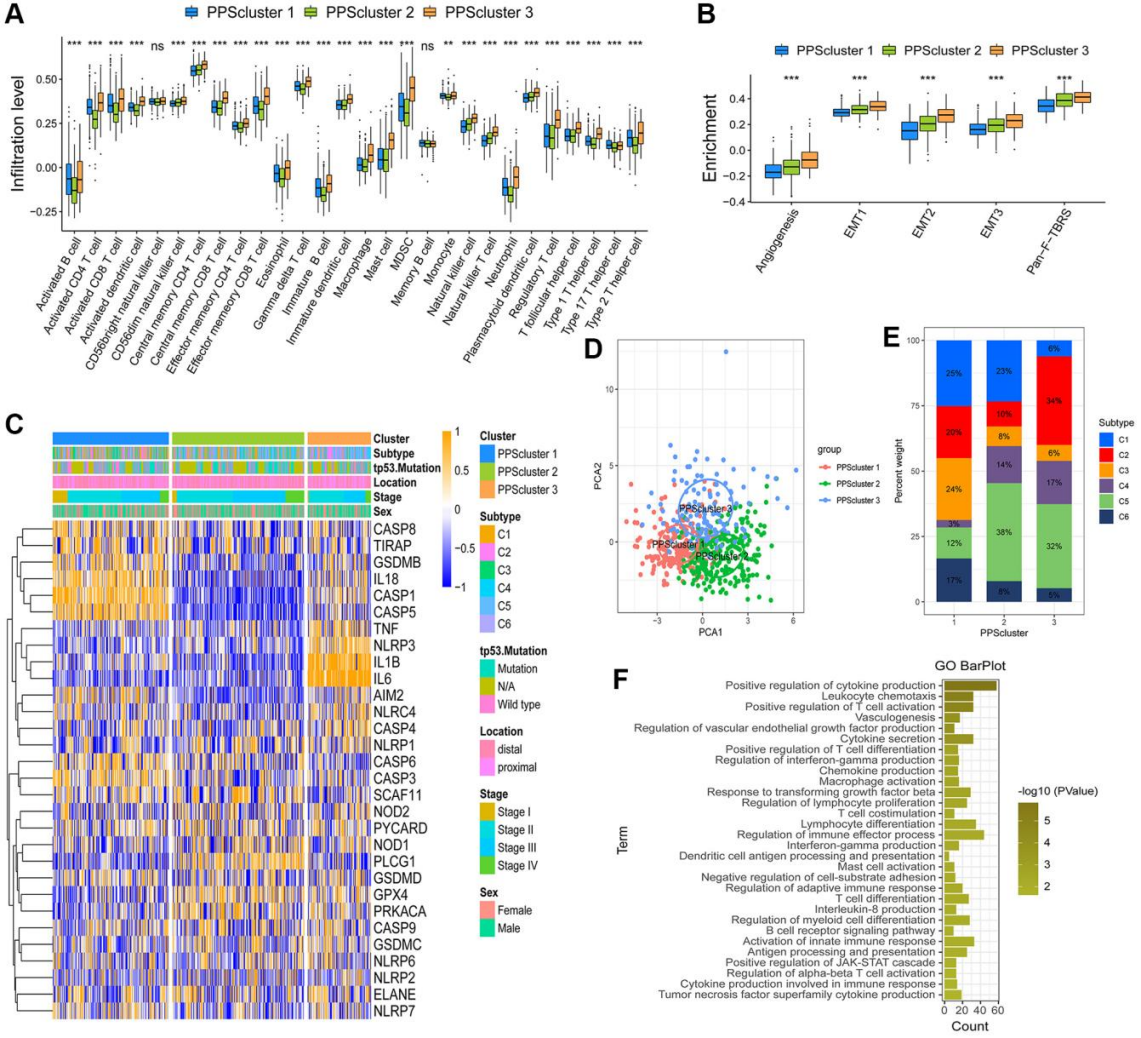
**TME cell infiltration characteristics in distinct pyroptosis patterns.** (**A**) The abundance of each TME infiltrating cell in three pyroptosis patterns (^*^P < 0.05; ^**^P < 0.01; ^***^P < 0.001). (**B**) Differences in stroma-activated pathways in three pyroptosis patterns (^*^P < 0.05; ^**^P < 0.01; ^***^P < 0.001). (**C**) Unsupervised clustering of 30 PRGs in the GSE39582 cohort. Clinicopathological information including tumor subtype, tp53 mutation, tumor location, tumor stage, and gender as well as the pyroptosis cluster, were shown in annotations above. Orange represented the high expression of genes and blue represented the low expression. (**D**) Principal component analysis of the transcriptome maps of the three pyroptosis patterns showed that there were significant differences among them. (**E**) The proportion of six molecular subtypes in GSE39582 cohort among three pyroptosis patterns. (**F**) The gene ontology enrichment analysis functionally annotates DEGs related to the pyroptosis patterns.

Furthermore, we used GSVA enrichment analysis to uncover the biological pathways underlying these three pyroptosis patterns. As a result, PPScluster-1 was enriched in activating T cell receptor signaling pathways, Toll like receptor signaling pathways and B cell receptor signaling pathways (Figure 2C). All of this indicated the fully activation of the immune. PPScluster-2 was prominently linked to the immune suppression biological process. While PPScluster-3 was markedly enriched in stromal and carcinogenic activation pathways such as ECM receptor interaction, cell adhesion and MAPK signaling pathways. However, subsequently analyses of TME revealed that PPScluster-3 was abundant in innate immune cell infiltration (Figure 3A). Additionally, cluster 3 showed significantly enhanced stroma activity (Figure 3B). But the patients in PPScluster-3 did not show a corresponding survival advantage (Figure 2B). Combining the distinct TME cell infiltration characterization of these three patterns with the novel concept of ‘immune contexture’, we classified PPScluster-1 as an immune-inflamed phenotype, characterized by immune cell infiltration and activation; PPScluster-2 as an immune-desert phenotype, characterized by the suppression of immunity; and PPScluster-3 as an immune-excluded phenotype, characterized by stromal activation (Figures 2C, 2D and 3A, 3B).

The specific correlation between each TME infiltration cell type and PRGs were explored (Supplementary Figure 1). High expression of TNF, NLRP3, NLRP1, NLRC4, IL-6, IL-1β, IL-18, CASP4 and AIM2 was significantly associated with enhanced immunocyte infiltration, whereas SCAF11, PLCG1 and CASP6 expression displayed a negative correlation with the immune infiltration level. From the above, we could speculate that pyroptosis patterns mediated by PRGs played indispensable roles in the immune regulation of TME.

### Pyroptosis patterns in GSE39582

Then, we analyzed the cohort GSE39582 (*n* = 585) to further explore these three phenotypes for their different clinical and biological characteristics. Excitingly, the unsupervised clustering results also obtained three similar patterns of pyroptosis from the dataset GSE39582 (Supplementary Figure 2A–2D and Figure 3C, 3D). Figure 3D showed, the pyroptosis transcriptional profile among these different patterns revealing a significant distinction. PPScluster-1 presented decreased expression of GPX4 and IL-6 genes, while varying increases in other PRGs. PPScluster-2 exhibited significant increases in the expression of GPX4, NOD1, PLCG1 and PRKACA genes. PPScluster-3 showed high expression of AIM2, CASP4, IL-1β, IL-6, NLRC4, NLRP3 and TNF genes (Supplementary Figure 2E). Marisa et al. (CIT cohort) stratified CC patients into six dominant molecular subtypes (CIN, CSC, dMMR, and KRASm), which were named after their main respective biological characteristics: C1, “CINImmuneDown”; C2, “dMMR”; C3, “KRASm” (for “KRAS-mutant”); C4 “CSC” (for “cancer stem cell”); C5, “CINWntUp”; and C6, “CINnormL” [23]. The prognosis of each of our six subtypes in the discovery set differed. C3 had a better prognosis, whereas C4 had a relatively poorer prognosis. The Oncotype DX score (an emerging prognostic classifier) classified 97% of the C4 samples as high risk. In our study, patients with the C4 subtype were predominantly clustered into PPScluster-2 and PPScluster-3, whereas patients with the C3 subtype were predominantly clustered into PPScluster-1 (Figure 3E). Moreover, the tp53 mutation that leads to a poor prognosis in patients was most prominent in PPScluster-2 (Figure 3C). This was consistent with our previous prognostic analysis of the three pyroptosis patterns. In addition, the prognostic analysis showed that compared to the shorter survival periods of PPScluster-2 and PPScluster-3, the survival period of PPScluster-1 was markedly prolonged (Supplementary Figure 2F).

### Generation of pyroptosis phenotype-related gene signatures and functional annotation

**Figure 4:**
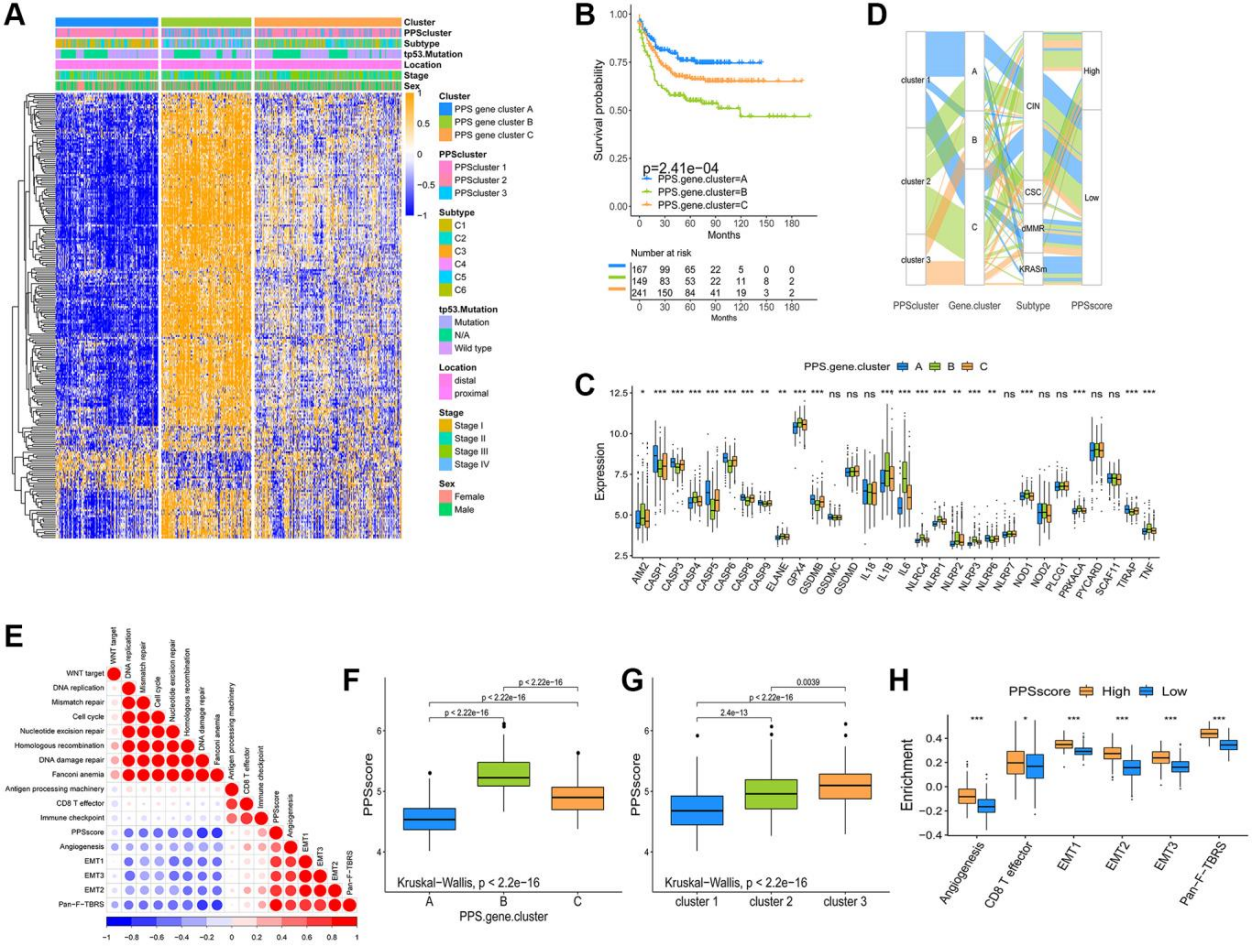
**Construction and analysis of pyroptosis signatures.** (**A**) Unsupervised clustering of overlapping pyroptosis phenotype-related genes in GSE39582 cohort to classify patients into three genomic subtype (PPS gene cluster **A**–**C**). Clinicopathological information such as tumor subtype, tp53 mutation, tumor location, tumor stage, and gender was used as patient annotations. (**B**) The survival curves of the pyroptosis phenotype-related gene signatures were shown using the Kaplan-Meier plotter. (**C**) The expression of 30 PRGs in three gene clusters (^*^P < 0.05; ^**^P < 0.01; ^***^P < 0.001). (**D**) The changes of PPSclusters, PPS gene clusters, tumor molecular subtypes and PPSscore were shown in the Alluvial diagram. (**E**) Correlations between PPSscore and the other gene signatures in GSE39582 CC cohort using Spearman analysis. Negative correlation was marked with blue and positive correlation with red. (**F**, **G**) The Kruskal-Wallis test was used to compare the statistical difference in PPSscore among three gene clusters and three PPSclusters. (**H**) Differences in stroma-activated pathways between high and low PPSscore groups ^(*^P < 0.05; ^**^P < 0.01; ^***^P < 0.001).

The limma package was used to detect 230 overlapping differentially expressed genes (DEGs) related to the pyroptosis phenotype (Supplementary Figure 2G). The clusterProfiler package was then used to perform Gene Ontology (GO) enrichment analysis on the DEGs. The results showed enrichment of biological processes such as leukocyte chemotaxis and positive regulation of T cell activation that are remarkably related to immunity, confirming once again that pyroptosis played a crucial role in the immune regulation in TME (Figure 3F). Then, we validated the regulatory mechanism involved. The method was to use unsupervised clustering analysis to classify patients into different genomic subtypes based on the 230 DEGs associated with the pyroptosis phenotype. Furthermore, the unsupervised clustering algorithm distinguished three pyroptosis genomic phenotypes, which were labeled PPS gene cluster A, PPS gene cluster B and PPS gene cluster C (Supplementary Figure 3A–3D, Figure 4A). Patients with a better prognosis for the C3 subtype were mostly concentrated in PPS gene cluster A. Tumors with PPS gene cluster C patterns had an abundant tp53 mutation subtype and were poorly differentiated. PPS gene cluster B identified patients with a poorer prognosis for the C4 subtype (Figure 4A). The survival analysis results of pyroptosis phenotype-related gene signatures were consistent with the preceding conclusions. CC patients in gene cluster A had a higher chance of survival. On the contrary, patients in gene cluster B had a worse prognosis. A moderate prognosis was observed in patients under gene cluster C (Figure 4B). In these three gene clusters, differences expression for 30 PRGs were observed (Figure 4C).

### Construction of the pyroptosis signature score and identification of its clinical relevance

We used published literature to extract cytokines and chemokines from them. We could explore the role of pyroptosis-related phenotypes in tumor immunity by studying their expression in these three gene clusters. TGFB2, SMAD9, PDGFRA, TGFBR2, TWIST1, ACTA2, COL4A1 and VIM are the genes involved in the transcription of the TGFb/EMT pathway. Immune checkpoint transcription genes include: PD-L1, CTLA-4, IDO1, LAG3, HAVCR2, PD-1, PD-L2, CD86, TIGIT, TNFRSF9. Immune activation genes include: IFNG, GZMB, CD8A, PRF1, GZMA, CXCL9, CXCL10. To better characterize the function of pyroptosis signature genes, we investigated known signature genes in CC patients (Supplementary Figure 3E). The findings showed that gene cluster B activated the matrix and promoted tumor progression. We also confirmed that most of the mRNAs that were significantly up-regulated in gene cluster B were related to the TGFb/EMT pathway, confirming that matrix activation was the main feature of this gene cluster. (Supplementary Figure 3F–3H). Finally, we obtained results that were consistent with the findings in Figure 4D, patients with the C4 “CSC” subtype in gene cluster B had a poor prognosis.

**Figure 5:**
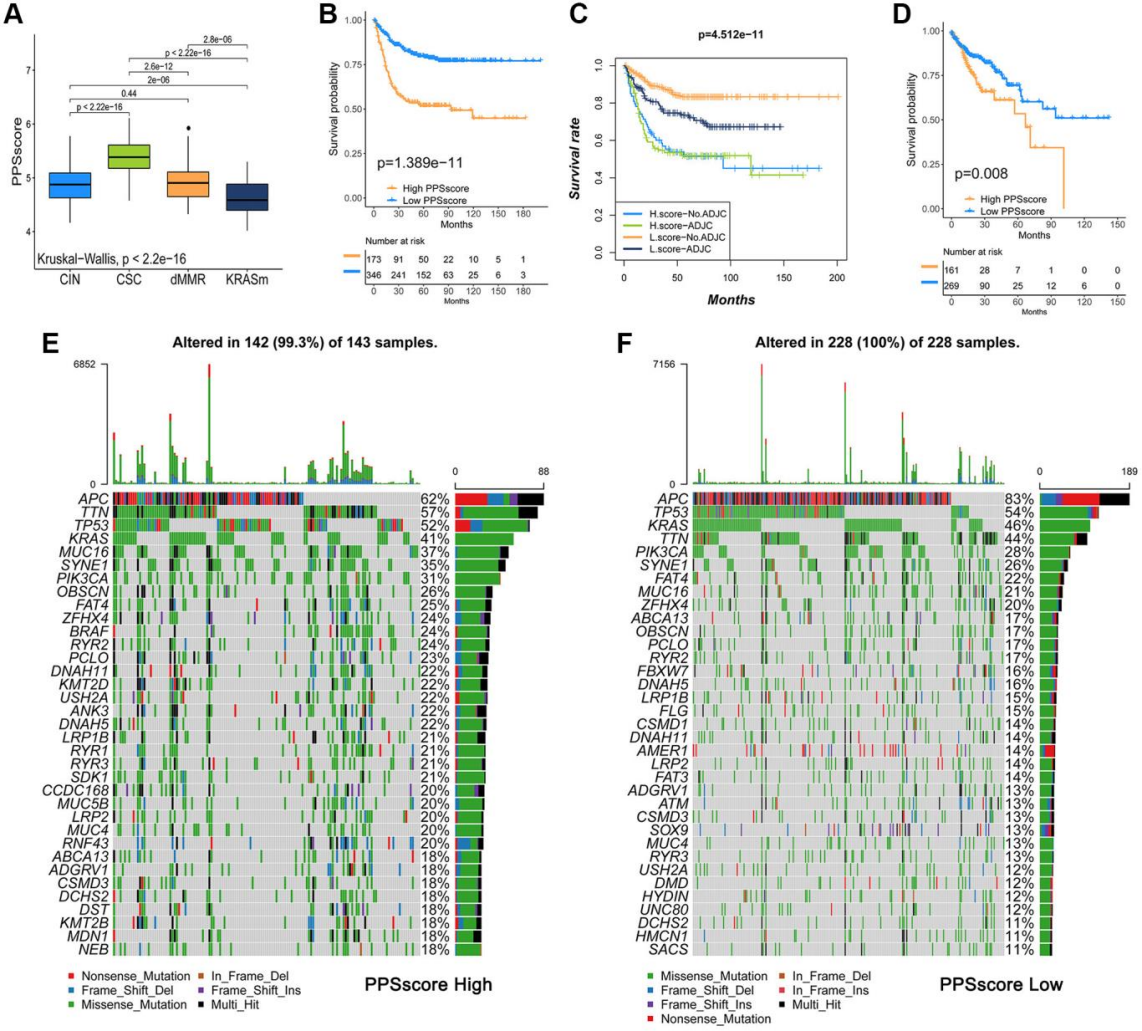
**Analysis of pyroptosis patterns characteristics and tumor somatic mutation.** (**A**) The Kruskal-Wallis test was used to compare the statistical differences of PPSscore among the four molecular subtypes. (**B**) Survival analyses for low and high PPSscore patient groups in GSE39582 using Kaplan-Meier curves. (**C**) Survival analyses for subgroup patients classified by PPSscore and treatment with adjuvant chemotherapy (ADJC) using Kaplan-Meier curves. (**D**) Survival analyses for low and high PPSscore patient groups in the TCGA-COAD cohort using Kaplan-Meier curves. (**E**, **F**) Tumor somatic mutation landscape in TCGA-COAD cohort were established according to high PPSscore (**E**) and low PPSscore (**F**). Each column represented individual patients. The upper barplot showed TMB. The right number indicated the mutation frequency in each gene. The right barplot showed the proportion of each variant type.

The findings above reveal that different pyroptosis patterns have a regulatory effect on the formation of TME landscapes, resulting in differences in patient prognosis. However, the analysis results of group patients could not, predict patterns of pyroptosis in individual patients to a certain extent. We constructed a pyroptosis signature score (PPSscore) using principal component analysis algorithms to quantify pyroptosis patterns for individual tumor patients with an immune response, considering the individual heterogeneity and complexity of pyroptosis patterns. The alluvial diagram was used to show the variation in characteristics of each tumor patient (Figure 4D). Furthermore, we investigated the potential relationship between known signatures and PPSscore (Figure 4E). The Kruskal-Wallis test revealed that PPSscore differed significantly between pyroptosis gene clusters. According to the median score results, gene cluster A scored the lowest, and gene cluster B scored the highest. When combined with the previous analysis, a low PPSscore may be linked to immune activation-related signatures, whereas a high PPSscore may be linked to stromal activation-related signatures (Figure 4F). It’s worth noting that PPScluster-3 had the significantly increased PPSscore than the other clusters, while PPScluster-1 had the lowest median score (Figure 4G). We further demonstrated that high scores were significantly associated with enhanced stromal pathways activation (Figure 4H). Furthermore, tumors with the C3- “KRASm” subtype had the lowest PPSscore compared to the other subtypes (Figure 5A). These results strongly suggested that PPSscore had certain advantages as an indicator for evaluating the pyroptosis patterns of individual tumors and could be used to further assess the characterization of TME in tumors.

To verify the predictive value of PPSscore in patient prognosis, we divided patients into a high and a low PPSscore group. Patients with a low PPSscore survived significantly longer (Figure 5B, *P* < 0.01). The independence was then calculated as a prognostic index for PPSscore in the CC sample. Considering the factors of patients’ adjuvant chemotherapy, gender, age, stage, and MMR status, multivariate Cox regression model analysis revealed that PPSscore was a reliable and independent prognostic biomarker for evaluating patient outcomes (HR 4.65 (2.98–7.2), Supplementary Figure 4A). Moreover, the ability of the PPSscore signature to predict the curative effect of adjuvant chemotherapy in CC was investigated. Patients with low PPSscore values who received adjuvant chemotherapy benefited significantly from the treatment. We also found that adjuvant chemotherapy did not affect the predictive ability of the PPSscore. That is, regardless of whether they received adjuvant chemotherapy or not, the low PPSscore group has a clear survival advantage (Figure 5C). Patients with lower scores were more likely to have survival advantage in the TCGA-COAD cohort (Figure 5D). In addition, we found that patients with higher pyroptosis values were more prone to disease progression (Supplementary Figure 4B). These findings suggest that the PPSscore had significant predictive value for clinical characteristics such as clinical stage and status.

### Characteristics of pyroptosis patterns in tumor somatic mutation

Tumor mutational burden (TMB) is one of the emerging biomarkers for predicting the response of tumor patients to immunotherapy. The efficacy of anti-PD-1/PD-L1 immunotherapy was significantly associated with patients' high TMB status. Therefore, we analyzed the distribution of somatic mutations in different PPSscores in the TCGA-COAD cohort. Figure 5E and 5F showed that the low PPSscore group had more extensive TMB than the high PPSscore group. However, the TMB quantification showed that tumors with a low PPSscore had a lower TMB (Supplementary Figure 5A). Besides, there was no significant correlation between PPSscore and TMB (Supplementary Figure 5B). Thus, the difference in PPSscore predicting the prognosis of CC patients may not be due to TMB. Further studies were needed to predict the clinical response and efficacy of TMB in patients with CC.

### The role of PPSscore in predicting immunotherapy

To further test the stability of the PPSscore model and validate its prognostic value for patients, we applied the PPSscore signature to other independent CC cohorts (GSE17536, GSE29621, GSE33113, GSE37892 and GSE38832) except GSE39582 (Supplementary Figure 6A–6E). The combined set of all GEO cohorts was validated (Supplementary Figure 6F). PPSscore was also tested for its ability to predict relapse-free survival (Supplementary Figure 6G). Subsequently, we continued to expand the predictive power of the PPSscore signature on 3-year and 5-year survival in CC patients (Supplementary Figure 6H and 6I). Using the ROC curve to evaluate the PPSscore was particularly advantageous in predicting the 3- and 5-year survival rates of CC patients.

**Figure 6:**
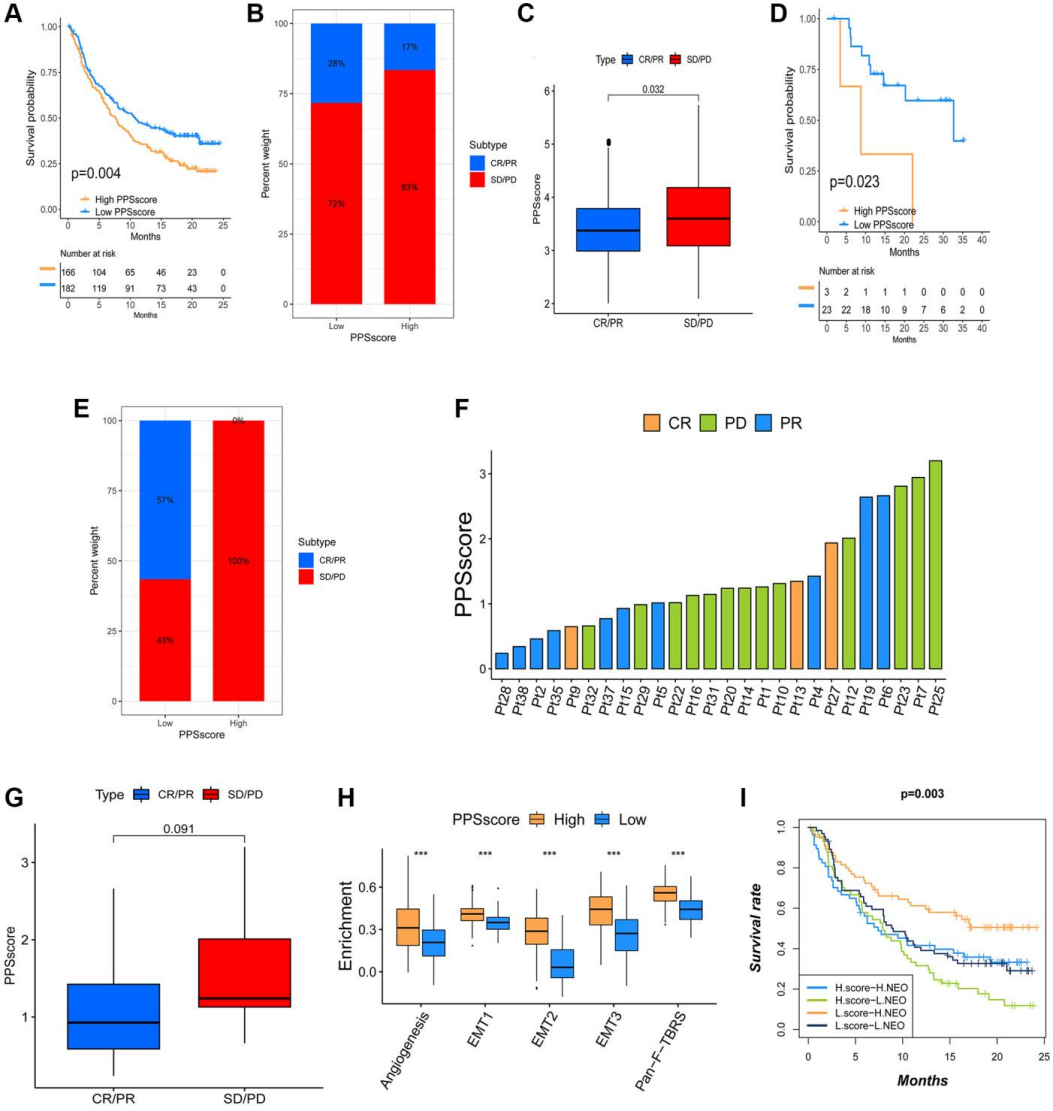
**The influence of distinct PPSscore on anti-PD-1/L1 immunotherapy.** (**A**) Survival analyses for low and high PPSscore patient groups in the anti-PD-L1 immunotherapy cohort using Kaplan-Meier curves. (**B**) The percent weight of patients with clinical response to anti-PD-L1 immunotherapy in low or high PPSscore groups. SD/PD, stable disease/progressive disease; CR/PR, complete response/partial response. (**C**) The distribution of PPSscore in distinct anti-PD-L1 clinical response groups. (**D**) Survival analyses for low and high PPSscore patient groups in the anti-PD1 immunotherapy cohort using Kaplan-Meier curves. (**E**) The percent weight of patients with clinical response to PD-1 blockade immunotherapy in low or high PPSscore groups. (**F**) The correlation of PPSscore with clinical response to anti-PD-1 immunotherapy. Pt, patients. PD, green; PR, blue; CR, orange. (**G**) The distribution of PPSscore in distinct anti-PD-1 clinical response groups. (**H**) Differences in stroma-activated pathways between high and low PPSscore groups in anti-PD-L1 immunotherapy cohort. The upper and lower ends of the boxes represented the interquartile range of values. The lines in the boxes represented the median value. The asterisks represented the statistical *P* value (^***^P < 0.001). (**I**) Survival analyses for patients receiving anti-PD-L1 immunotherapy classified by both PPSscore and neoantigen burden using Kaplan-Meier curves.

Immunotherapy has gained wide attention for cancer therapy. Therefore, we investigated whether pyroptosis pattern signals could predict patients’ responses to immune checkpoint blockade therapy. We found that low PPSscore patients had a significant survival advantage in both the anti-PD-L1 cohort (IMvigor210) and anti-PD-1 (GSE78220) cohort (Figure 6A–6G). Patients with low PPSscore who receiving anti-PD-1/L1 immunotherapy had a significant clinical response and therapeutic advantage over these with high PPSscore. Further studies showed that TME stroma was significantly activated with a high PPSscore. We speculated that these processes might mediate tumor immune tolerance mechanisms, resulting in a poor prognosis in tumor patients (Figure 6H). Current studies have shown that tumor neoantigen burden was the important factor affecting the effect of immunotherapy. Through comparative analysis, we finally determined the conditions under which patients could benefit from survival: low PPSscore and high neoantigen burden (Figure 6I). When combined with the preceding evidence, quantification of pyroptosis patterns (PPSscore) was an independent and promising biomarker, particularly for evaluating the clinical efficacy of immunotherapy and predicting patients’ prognosis. The pyroptosis patterns and characteristics established in this study could aid in the clinical prediction of patients’ responses to anti-PD1/L1 immunotherapy, and provide a foundation for CC patients’ prognosis.

## DISCUSSION

Pyroptosis, a new form of programmed cell death, is characterized by rapid plasma-membrane rupture and the release of proinflammatory intracellular contents [24]. The activated caspases cleave the hinge region between the N- and C-terminal domains of genes, releasing the lethal segment and causing pyroptosis [25, 26]. Pyroptosis can influence the TME antitumor immune response [27–29]. For instance, CD8^+^ T cells and NK cells in the TME can trigger tumor clearance via the GSDMB granzyme A axis, which is aided by IFN-γ [20]. Furthermore, colocalization of GSDMD and granzyme B (an enzyme capable of cleaving GSDME) was observed near immune synapses, and GSDMD deficiency reduced CD8^+^ T cells cytolytic capacity [22]. ICIs are severely limited in most cancer types because they do not work in all cancer patients [30]. Tumors resistant to immune checkpoint inhibitors are deemed “cold”: their TME could be changed, which involved a series of immune tolerance mechanisms, including the recruitment of immunosuppressive cells, and the release of immunosuppressive factors. However, a previous study found that pyroptosis “heats” anticancer immunity [31]. As previously stated, PRGs may predict survival and influence immunotherapy. Nonetheless, characterizations of panoramic TME infiltration mediated by integrated roles of PRGs in CC are not entirely clear. We identified three pyroptosis patterns in TME cell infiltration, which will help us focus on the role of pyroptosis in the formation of TME and immunity, providing a solid foundation for developing immunotherapy strategies.

In this study, 33 PRGs were integrated from previous reviews into six GEO datasets (GSE39582, GSE38832, GSE37892, GSE33113, GSE29621, and GSE17536) of the CC in our study, and eventually, 30 PRGs were identified. Based on these identified genes, we obtained three distinct pyroptosis patterns: PPScluster-1, PPScluster-2, and PPScluster-3. These patterns had different survival probabilities and distinct TME cell infiltration characterization. The results showed that the PPScluster-1 pattern occupied a particularly significant survival advantage. Simultaneously, GSVA enrichment analysis revealed that PPScluster-1 was markedly enriched in immune activation pathways. However, PPScluster-2 (immune suppression) and PPScluster-3 (immune-excluded) patients had a lower survival probability than CC patients in the PPScluster-1 pattern. It appears that pyroptosis plays a vital role in the immune regulation of the TME. Interestingly, the TME cell infiltration analyses revealed that PPScluster-3 (immune-excluded phenotype) was remarkably rich in innate immune cell infiltration (Figure 3A), but patients with this pattern did not have a significantly improved prognosis (Figure 2B). Previous studies have shown that immune cells are abundant in tumors with an immune-excluded profile. What is noteworthy is that these immune cells are retained in the stroma surrounding tumor cell nests rather than penetrating the tumor parenchyma [15, 32, 33]. Clinical responses are uncommon when stroma-associated T cells show no evidence of infiltration [14]. GSVA analysis showed that the cluster 3 pattern was significantly related to stromal activation. T cell suppression was thought to activate stroma in the TME. Therefore, we speculated that the antitumor effect in cluster 3 was mediated by stromal activation, resulting in immune T cell suppression. Furthermore, the matrix activity in cluster 3 was significantly enhanced, such as the activation of EMT and TGFb, confirming our speculation. Therefore, this study confirmed that the results of the immune phenotype classification generated by our clustering results were meaningful.

Furthermore, DEGs were found in different pyroptosis phenotypes. Subsequently, GO enrichment analysis revealed that the DEGs in different clusters were linked to immune-related pathways. These findings confirmed that pyroptosis played a significant role in the immune regulation in the TME of CC. Similarly, the unsupervised clustering algorithm identified three distinct pyroptosis genomic phenotypes based on the obtained DEGs. The current study’s survival analysis results revealed different survival probabilities in the three gene clusters. As previously stated, it confirmed that distinct pyroptosis-related patterns did exist in CC and that pyroptosis played a key role in shaping distinct TME landscapes.

However, in the clinic, CC was found to have some contradictions, “the first neoplasia found to be under immunosurveillance and the last one to respond to immunotherapy” [34]. The reason may be due to the individual heterogeneity of CC, characterized by different biomolecular, anatomical, and gene signatures. Existing differences influence tumor behavior [35, 36]. An immune response is not assured in every individual, even if they belong to the immune-inflamed phenotype. Patients may vary in their responses. Therefore, we developed the ‘PPSscore’ scoring system to quantify individual pyroptosis patterns in CC patients. The apparent superiority of the PPSscore is that it considers the heterogeneity of CC patients and can link pyroptosis to prognosis. The results revealed that PPScluster-1 had the lowest median PPSscore. Moreover, in this study’s anti-PD-L1 and anti-PD-1 cohorts, patients with a low PPSscore had a significant survival advantage. Our findings strongly demonstrated that the PPSscore could be a promising way of evaluating individual tumor pyroptosis patterns and determining tumor immune phenotypes.

## CONCLUSIONS

We evaluated the pyroptosis patterns of 1689 CC patients using 30 PRGs. The three patterns identified in our study were highly consistent with the three known immune phenotypes of tumors, including immune-excluded, immune-inflamed, and immune-desert phenotypes. We also confirmed that distinct pyroptosis patterns were important in immunoregulation and the development of TME diversity and complexity. Quantifying pyroptosis patterns in individual tumors could improve our understanding of TME infiltration characterization and effectively predict patient clinical response to immunotherapy. This current research might provide a profound approach to discovering effective immunotherapeutic strategies.

## MATERIALS AND METHODS

### Data acquisition and preprocessing of CC datasets

Public gene-expression data and clinical annotation of CC were obtained from GEO (https://www.ncbi.nlm.nih.gov/geo/) and TCGA (https://cancergenome.nih.gov/) database. Patients lacking survival information were excluded from this study. In total, 1689 patients from 7 eligible CC cohorts were enrolled. These cohorts included GSE39582 (*n* = 585), GSE38832 (*n* = 122), GSE37892 (*n* = 130), GSE33113 (*n* = 96), GSE29621 (*n* = 65), GSE17536 (*n* = 177) and the Cancer Genome Atlas-Colon Adenocarcinoma (TCGA-COAD, *n* = 514). The basic information from the CC datasets included in the group was summarized in Supplementary Table 1. We used the raw “CEL” files for the microarray data from Affymetrix^®^. For background adjustment and quantile normalization, we performed a robust multi-array averaging method of the affy and simpleaffy packages. TCGA RNA sequencing data (FPKM value) were downloaded from the Genomic Data Commons (GDC, https://portal.gdc.cancer.gov/) using the R package TCGAbiolinks. Subsequently, FPKM values were transformed into transcripts per kilobase million (TPM) values. The ComBat method of the “SVA” R package was applied to correct the batch effects from non-biological technical biases. The somatic mutation data was obtained from the TCGA database.

### Unsupervised clustering for 30 PRGs

Initially, we extracted 33 PRGs from previous reviews. In our study, these genes were integrated into six CC GEO datasets. Finally, a set of 30 genes was extracted for identifying different pyroptosis forms mediated by these genes. We used unsupervised clustering analysis to differentiate pyroptosis patterns and classify patients based on PRGs expression. The consensus ClusterPlus package was used to execute the clustering algorithm, and ensure the stability of classification through 1000 times repetitions.

### Gene set variation analysis (GSVA) and functional enrichment analyses

The GSVA was then used to further investigate the differences in biological processes between different pyroptosis patterns, we subsequently conducted the GSVA. “c2.cp.kegg.v6.2.symbols” gene sets were downloaded from the MSigDB database and an adjusted *P* < 0.05 was considered statistically significant. The R package ‘clusterProfiler’ was used to implement functional annotation for PRGs. FDR< 0.05 was set as the cutoff value.

### Estimation of immune cell infiltration in the CC TME

The single-sample gene set enrichment analysis (ssGSEA) algorithm was used to assess the status of immune cell infiltration in the TME. The recent study provided the special feature gene set for identifying each TME infiltration immune cell type. A ssGSEA enrichment score calculated the relative abundance of TME-infiltrated cells in each sample.

### Identification of DEGs among distinct pyroptosis patterns

This study’s above unsupervised clustering analysis results in this study classified CC patients into three distinct pyroptosis patterns (PPScluster-1, PPScluster-2 and PPScluster-3), from which we determined pyroptosis-related DEGs among these three distinct pyroptosis phenotypes. DEGs in CC samples were evaluated using the empirical Bayesian approach of the limma R package. The filtering criteria for DEGs were set to an adjusted *P-*value < 0.001.

### Establishment of pyroptosis gene signature

Considering the individual heterogeneity, we constructed PPSscore, a set of scoring systems, for evaluating pyroptosis forms in individual tumors. The DEGs identified from the three distinct pyroptosis clusters were firstly normalized for all GSE39582 samples, and the overlapping genes were extracted. By analyzing DEGs, an unsupervised clustering method was developed to classify patients into different groups. Then, for each gene, we used a univariate Cox regression model to perform a prognostic analysis. The genes with *P* < 0.05 were extracted for further analysis. We then conducted the LASSO Cox regression algorithm to construct a pyroptosis- relevant gene signature. The PPSscore was defined as follows:

$$\text{PPS }score=\sum_{i=1}^{n}Coefi\times Expri$$

where Expri represented the signature genes and the LASSO Cox regression provided the Coefi coefficient.

### Collection of two immunotherapeutic cohorts information

We discovered two immunotherapeutic cohorts after systematically searching the ICB gene expression profiles. For further analysis, the gene expression profiles of advanced urothelial cancer patients treated with atezolizumab (anti-PD-L1 antibody, IMvigor300 cohort) and metastatic melanoma patients treated with pembrolizumab (anti-PD-1 antibody, GSE78220 cohort) were all transformed into the TPM value for further analysis.

### Statistical analysis

R version 3.6.1 and the appropriate packages were used for statistical analysis. The correlation coefficients between TME infiltrating immune cells and pyroptosis gene expression were computed using Spearman and distance correlation analyses. One-way ANOVA and the K-W tests were used to compare differences between groups. Survminer R software package was used to determine the cutoff points for each subgroup of data sets. The “surv-Cutpoint” function was used to find the maximum rank statistic. Patients were divided into high and low PPSscore groups according to the maximum log-rank statistics. The Kaplan-Meier method was used to generate survival curve for prognostic analysis. A multivariate Cox regression model was used to determine the independence of the variables. Especially for patients with detailed clinical data.The results visualization for PPSscore in GSE39582 cohort was achieved through the forestplot R package. The mutational landscape in CC patients with high and low PPSscore subtypes in the TCGA-COAD cohort was plotted using the waterfall function of the R ‘maftools’ package. The CNV landscape of the 30 PRGs was depicted using the RCircos R package. A two-sided *P*-value <0.05 was considered statistically significant.

## Supplementary Materials

Supplementary Figures

Supplementary Table 1
