# Supplementary material for: Pyroptosis patterns of colon cancer could aid to estimate prognosis, microenvironment and immunotherapy: evidence from multi-omics analysis
Source: Aging (Albany NY). 2022 Sep 23;14(18):7547–67. doi: 10.18632/aging.204302 (PMC9550258; doi:10.18632/aging.204302)
Supplement: Supplementary Figures [file aging-14-204302-s001.pdf]

SUPPLEMENTARY FIGURES

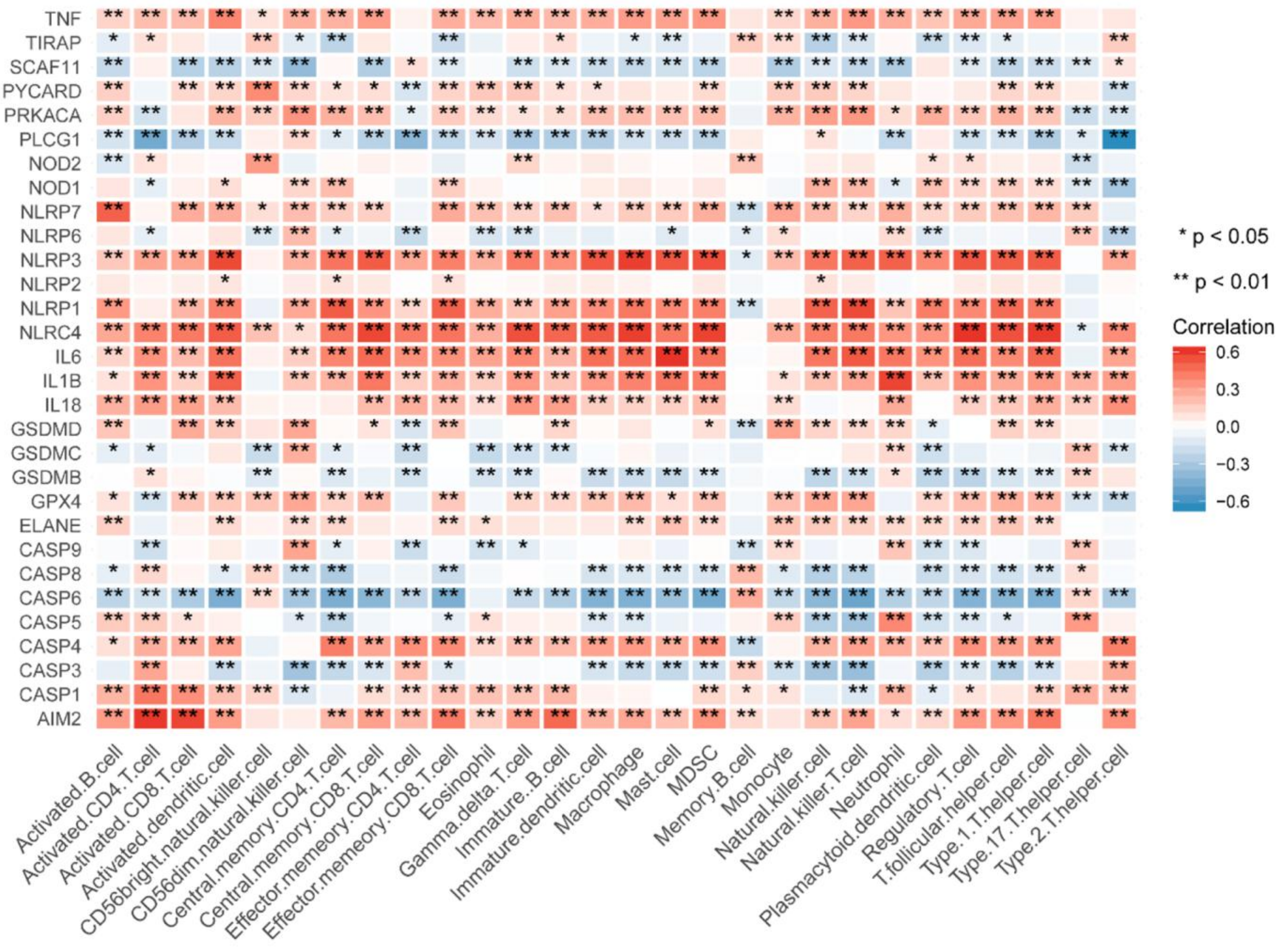

Supplementary Figure 1. Spearman analysis was used to obtain the correlation between each TME infiltrating cell type and PRGs. Blue represented negative correlation and red represented positive correlation.

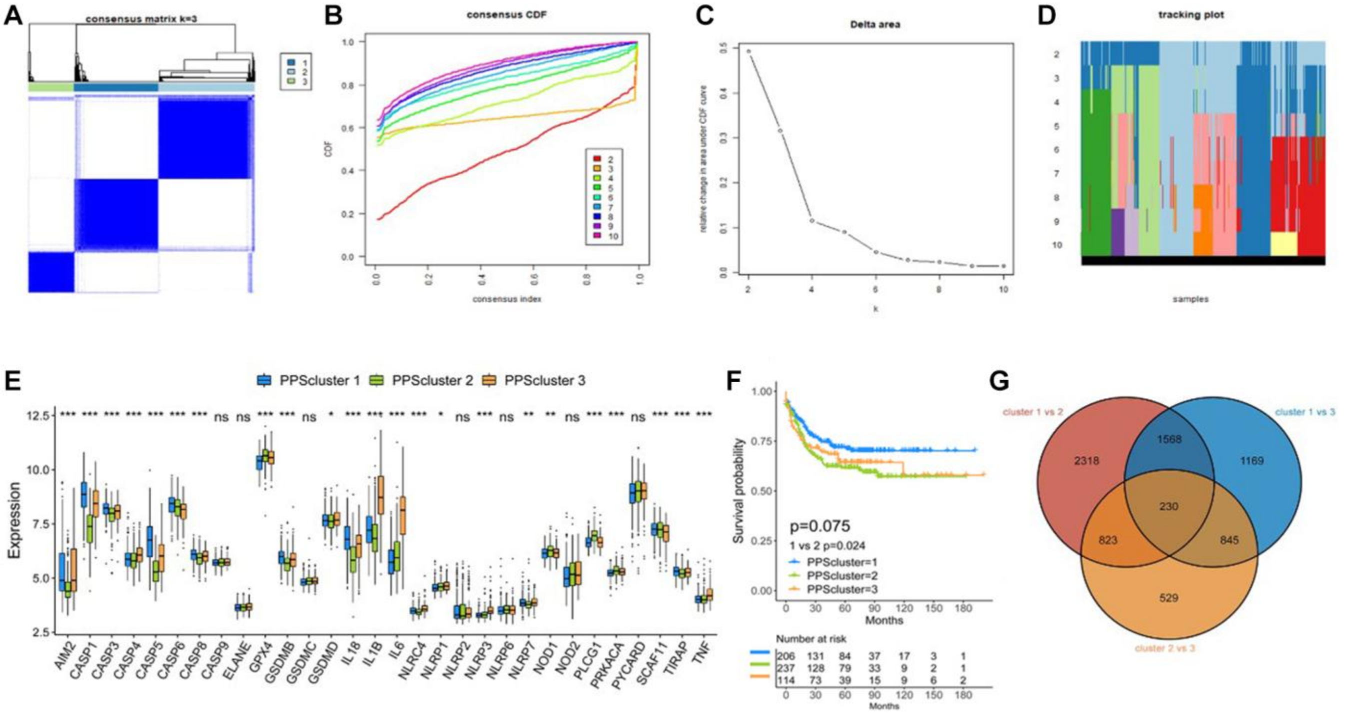

Supplementary Figure 2. Unsupervised clustering of 30 PRGs in GSE39582 CC cohort. (**A**) The heatmap of consensus matrices for GSE39582 CC cohort (k = 3). (**B**) Empirical cumulative distribution function (CDF) plots displayed consensus distributions for each k. (**C**) The delta area score (y-axis) indicated the relative increase in cluster stability. (**D**) The item tracking plot showed the consensus cluster of items (in columns) at each k (in rows). (**E**) The expression of 30 PRGs in the three PPSclusters ($^{*}P < 0.05$; $^{**}P < 0.01$; $^{***}P < 0.001$). (**F**) Survival analyses for the three pyroptosis patterns in GSE39582 using Kaplan-Meier curves. (**G**) 230 overlapping DEGs related to pyroptosis phenotype were shown in Venn diagram.

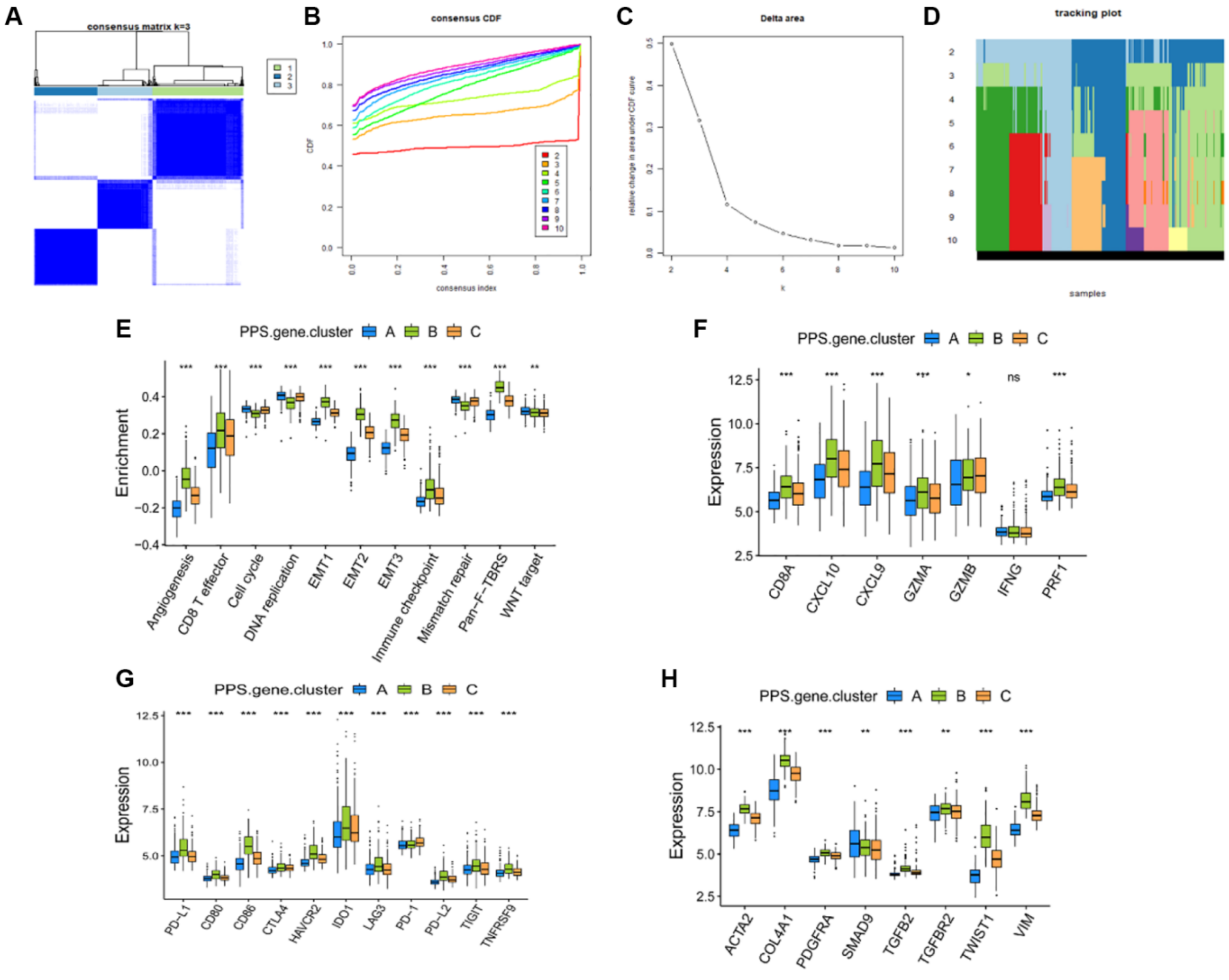

Supplementary Figure 3. Characteristics of cytokine transcriptome, chemokine transcriptome and known signatures in distinct gene clusters. (**A**) The heatmap of consensus matrices for GSE39582 CC cohort (k = 3). (**B**) Empirical cumulative distribution function plots displayed consensus distributions for each k. (**C**) The delta area score (y-axis) indicated the relative increase in cluster stability. (**D**) The item tracking plot showed the consensus cluster of items (in columns) at each k (in rows). (**E**) Difference in the expression of known signatures including stromal-activation related signatures, tumor-promotion related signatures and immune-activation related signatures among three gene clusters ($^{*}P < 0.05$; $^{**}P < 0.01$; $^{***}P < 0.001$). (**F**) Difference in the immune-activation related gene expression among three gene clusters. (**G**) Difference in the immune-checkpoint related gene expression among three gene clusters. (**H**) Difference in the TGFβ- EMT pathway-related gene expression among three gene clusters.

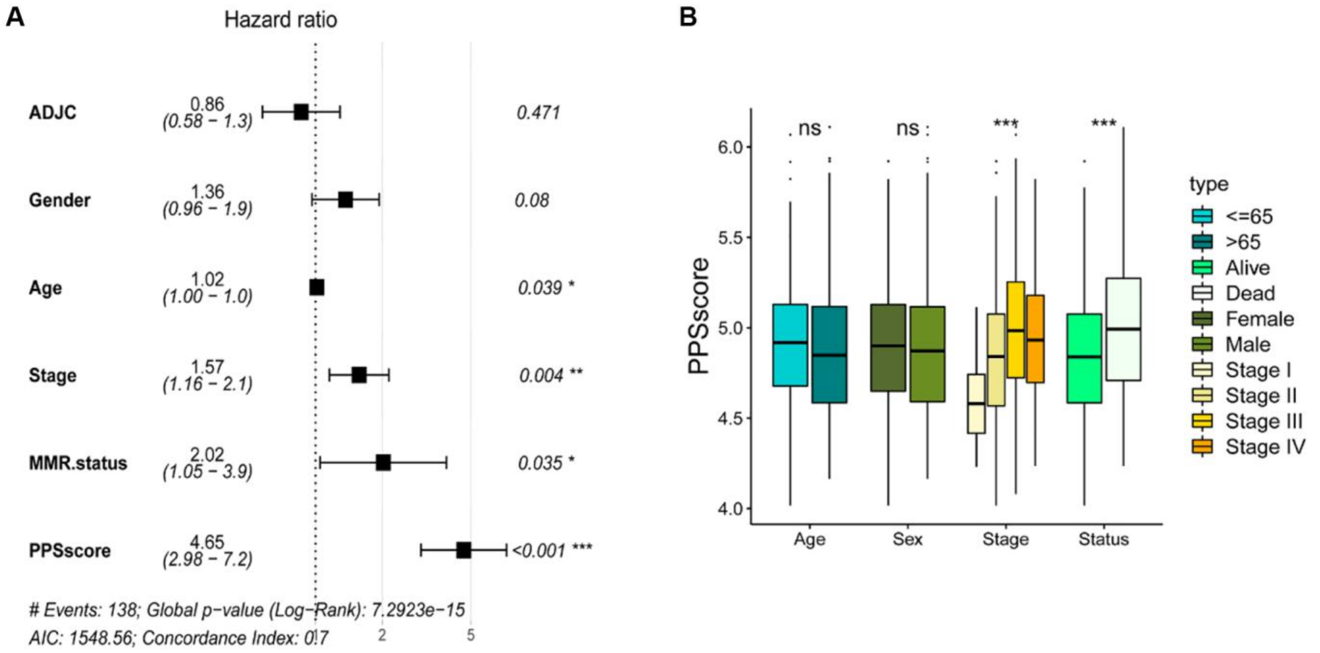

Supplementary Figure 4. The prognostic value of PPSscore and the correlation between the clinicopathological features and PPSscore. (**A**) Multivariate Cox regression analysis for PPSscore in GSE39582 cohort shown by the forest plot. (**B**) Difference in PPSscore among distinct clinical subgroups in GSE39582 cohort. ADJC, adjuvant chemotherapy.

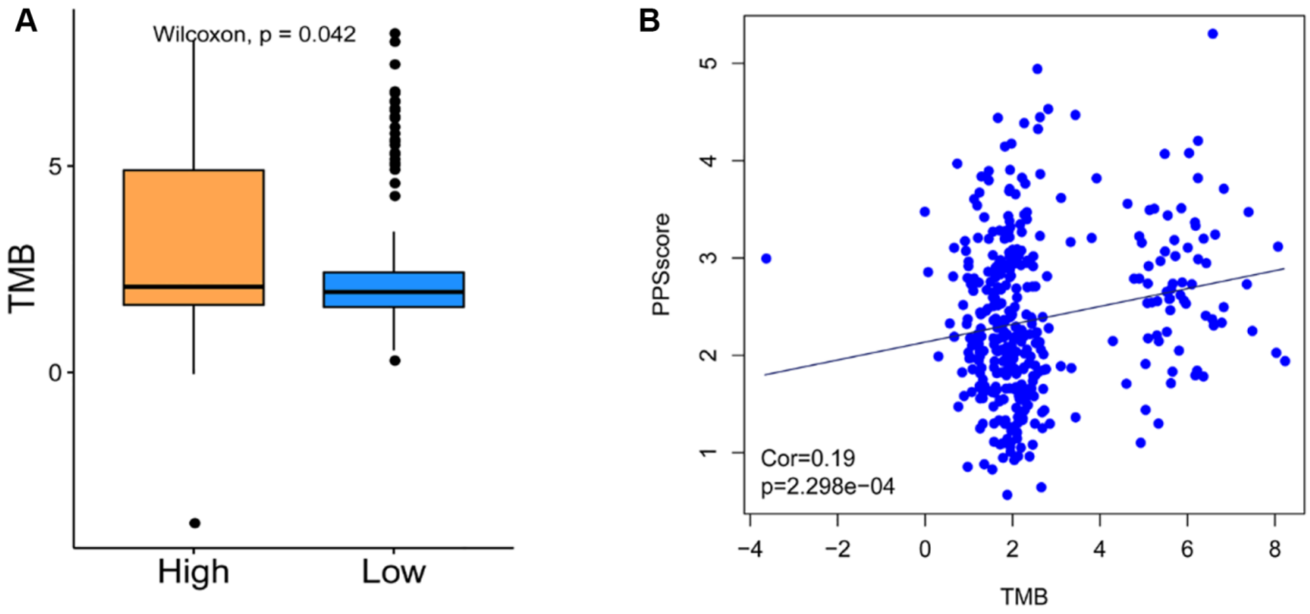

Supplementary Figure 5. The relationship between tumor mutation burden (TMB) and PPSscore. (**A**) The distribution of tumor mutation burden (TMB) in distinct PPSscore groups (P = 0.042, Wilcoxon test). (**B**) There was a positive correlation between TMB and PPSscore (r = 0.19, $P < 0.001$).

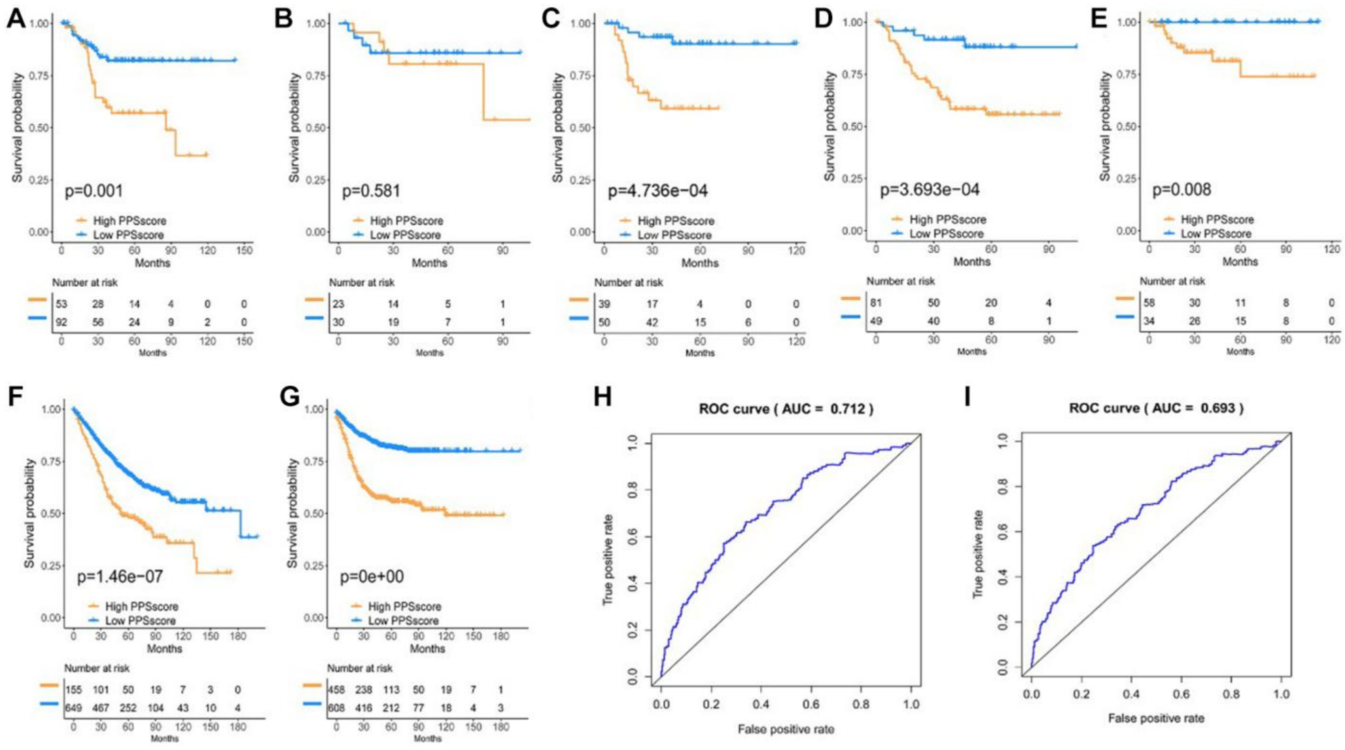

Supplementary Figure 6. The prognostic value of PPSscore in CC cohorts. Survival analyses for low and high PPSscore patient groups in (**A**) GSE17536, (**B**) GSE29621, (**C**) GSE33113, (**D**) GSE37892 and (**E**) GSE38832 using Kaplan-Meier curves. (**F**) Overall survival analysis of PPSscore in all GEO CC cohorts. (**G**) Relapse-free survival analysis of PPSscore in all GEO CC cohorts. (**H**) The predictive power of the PPSscore signature on 3-year survival in GSE39582 cohort (AUC = 0.712). (**I**) The predictive power of the PPSscore signature on 5-year survival in GSE39582 cohort (AUC = 0.693).
