# Supplementary material for: Pyroptosis patterns of colon cancer could aid to estimate prognosis, microenvironment and immunotherapy: evidence from multi-omics analysis
Source: Aging (Albany NY). 2022 Sep 23;14(18):7547–67. doi: 10.18632/aging.204302 (PMC9550258; doi:10.18632/aging.204302)
Supplement: Supplementary Table 1 [file aging-14-204302-s002.pdf]

SUPPLEMENTARY TABLE

Supplementary Table 1. The GEO and TCGA datasets included in this study.

Accession number	Source	Number of patients	Survival
TCGA: COAD	Illumina RNAseq	514	OS
GEO: GSE39582	Affymetrix Human Genome U133 Plus 2.0 Array	585	RFS/OS
GEO: GSE38832	Affymetrix Human Genome U133 Plus 2.0 Array	122	RFS/OS
GEO: GSE37892	Affymetrix Human Genome U133 Plus 2.0 Array	130	RFS
GEO: GSE33113	Affymetrix Human Genome U133 Plus 2.0 Array	96	RFS
GEO: GSE29621	Affymetrix Human Genome U133 Plus 2.0 Array	65	RFS/OS
GEO: GSE17536	Affymetrix Human Genome U133 Plus 2.0 Array	177	RFS/OS
